# Stellate ganglion block treats posttraumatic stress: An example of precision mental health

**DOI:** 10.1002/brb3.1807

**Published:** 2020-08-28

**Authors:** James H. Lynch

**Affiliations:** ^1^ Department of Military and Emergency Medicine Uniformed Services University of the Health Sciences Bethesda MD USA

**Keywords:** posttraumatic stress, posttraumatic stress disorder, precision medicine, stellate ganglion block, sympathetic blockade, trauma‐focused therapy

The U.S. Precision Medicine Initiative (PMI), launched in 2015, is a nationwide initiative to move away from “one‐size‐fits‐all” medicine and instead to tailor treatment strategies to a patient's unique characteristics (HealthIT.gov). The long‐term goals of this effort focus on bringing precision medicine to all areas of health on a large scale (U.S. National Library of Medicine, 2020). Psychiatry is exceptionally complex compared to many other medical fields where precision diagnostics rely on quantified laboratory values, imaging findings, cytology, etc. Psychopathology is primarily diagnosed by an expert clinician's judgment applying criteria from the Diagnostic and Statistical Manual of Mental Disorders (DSM‐5) (American Psychiatric Association, 2013). Over recent years, some have questioned the utility of such psychiatric taxonomy in present day clinical treatment (Dalgleish, Black, Johnston, & Bevan, 2020). These diagnostic aspects complicate the goal of achieving precision medicine for mental illness. Moving forward, reframing mental illness as disorders of brain functioning may facilitate these discussions (Fernandes et al., 2017). Many will associate the term “precision medicine” in psychiatry with current efforts to discover genetic variables and biotypes for mental illness. I offer an alternate use of the term here. A procedure called stellate ganglion block used for posttraumatic stress symptoms provides us with a contemporary example of delivering “precision mental health” now without waiting for future research to provide a more complete understanding of the pathophysiology of mental disorders (DeRubeis, 2019).

## 1 DEFINING THE PROBLEM

Using DSM‐5 criteria, there are over 636,000 possible combinations of symptoms that qualify for the diagnosis of posttraumatic stress disorder (PTSD). For comparison, major depressive disorder has 227 possible combinations, and panic disorder has 23,442 combinations (Galatzer‐Levy & Bryant, 2013). It is challenging to study such a heterogeneous condition and draw logical conclusions about which treatments are effective for the vast array of symptom combinations. This may explain why it is difficult to determine which PTSD treatments are superior. It may also explain why many current standard PTSD therapies demonstrate somewhat disappointing results and weak effect sizes when evaluated in large clinical trials (Lewis, Roberts, Andrew, Starling, & Bisson, 2020; Watts et al., 2013). While scientifically sound and widely accepted, using aggregate outcomes such as change in mean Clinician‐Administered PTSD Scale (CAPS) to determine if a treatment is superior to placebo provides an incomplete answer to the question of “What works for whom?” particularly when applied to the patient sitting in front of you with one of 636,000 posttraumatic symptom combinations (DeRubeis, 2019).

Worth mentioning are the countless undiagnosed individuals in need of mental health care who fall short of DSM‐5 criteria for PTSD despite suffering from significant posttraumatic symptoms (Kotov, Krueger, & Watson, 2018). Based on DSM‐5, there are over 100,000 combinations of PTSD symptoms that do not meet full diagnostic criteria despite having one or more symptoms in all categories (Galatzer‐Levy & Bryant, 2013). This implies that there are many patients who may benefit from therapies that target the *symptoms* of PTSD rather than purely treating the *diagnosis*.

In line with the goals of the Precision Medicine Initiative, some initial gains have been made in the field of genomics, but we may be years away from their practical application in mental health (Stein & Smoller, 2018). It is certainly likely that someday we will personalize treatment for all patients with mental disorders based on a complex analysis of “biotypes” (Stanford Medicine Department of Psychiatry and Behavioral Sciences). In the meantime, the science exists today to improve our approach to a disorder that affects over 10 million Americans with PTSD by applying more precise diagnostics and neuroscience‐informed treatment (American Psychiatric Association, 2020). The goal is the same—to more precisely target our patients’ distinct symptomatology.

## 2 THE UNDERLYING BRAIN CIRCUITRY MALFUNCTION

While not completely elucidated presently, several maladaptive biologic processes likely contribute to different posttraumatic symptoms. Some PTSD symptoms are caused by a stimulated sympathetic nervous system including hyperarousal symptoms such as irritability, angry outbursts, and heightened startle reflex. The physical symptoms which accompany reexperiencing traumatic events including racing heart, sweating, and increased body tone are sympathetically driven. This psychological overlap with the neuroendocrine system should be understood and exploited to focus therapies for PTSD which target hyperarousal when present.

For many trauma victims, hyperarousal is a predominant PTSD feature. This cluster of symptoms has been attributed to a type of “dysfunctional sympathetic tone” describing a process where somatic response to stimuli is inappropriately amplified (Mulvaney et al., 2014). Many of these physical symptoms of PTSD serve as barriers to effective psychotherapy. Previous research has identified several predictors of poor PTSD treatment trajectories such as comorbid depression and substance abuse. Exaggerated hyperarousal is also an independent predictor of nonresponse to treatment (Averill, Averill, Fan, & Abdallah, 2020). Despite large decreases in clinician‐rated symptoms, nearly half of patients completing cognitive behavioral therapy (CBT) for PTSD still manifest clinically significant insomnia, anger, and irritability (Zayfert & DeViva, 2004). PTSD patients with high levels of hyperarousal at the onset of therapy may require additional treatment (Stein, Dickstein, Schuster, Litz, & Resick, 2012).

Hyperarousal symptoms can be specifically addressed in a variety of ways (e.g., meditation) (Crawford, Talkovsky, Bormann, & Lang, 2019). Some treatments targeting an overstimulated sympathetic nervous system may have undesirable side effects. This is particularly true with some pharmacologic agents (e.g., benzodiazepines) and can contribute to noncompliance and dropout (Guina, Rossetter, DeRhodes, Nahhas, & Welton, 2015). Given the known difficulty treating hyperarousal in PTSD, clinicians should strongly consider precision treatment plans tailored to the individual patient.

## 3 INTEGRATING BRAIN SCIENCE WITH TECHNOLOGY

Advanced neuroimaging techniques such as functional magnetic resonance imaging (fMRI) have identified several neuronal circuits relevant to the pathophysiology of PTSD. Prominent findings in PTSD include (a) hyperactivation of the amygdala and dorsal anterior cingulate cortex (dACC), (b) hypoactivation of the ventromedial prefrontal cortex, and (c) atrophy of the hippocampus (Kamiya & Abe, 2020). A network‐based neurobiologic model defines a central autonomic network composed of three interconnected subnetworks located in different regions of the brain—the salience, central executive, and default mode networks. The core structures of the salience network are the amygdala, insula, and dACC, which are associated with hyperarousal and found to be hyperactive on fMRI in PTSD patients (Menon, 2011).

The interconnections between these specific regions of the brain's central autonomic network are complex. However, the cervical sympathetic trunk which runs deep in the neck has been described as an “anatomic funnel through which all sympathetic fibers must flow on their way to the head, neck, and thorax” (Moore, 1954). The stellate ganglion, located along the cervical sympathetic trunk at the level of C7‐T1, provides a connection to the hypothalamus and central nucleus of the amygdala as well as the insular cortex (Westerhaus & Loewy, 2001). This neural circuit connecting the brain to the body provides a precise anatomic target to address the physical symptoms of hyperarousal.

## 4 NEUROSCIENCE‐INFORMED TREATMENT

For over one hundred years, a simple, safe procedure called stellate ganglion block (SGB) has been used successfully to treat a variety of sympathetically modulated pathologies ranging from chronic regional pain syndrome (CRPS) to postherpetic neuralgia (Imani, Hemati, Rahimzadeh, Kazemi, & Hejazian, 2016; Moore, 1954; Moore & Bridenbaugh, 1956; Summers & Nevin, 2017). Stellate ganglion block is an injection of local anesthetic in the neck to temporarily block the cervical sympathetic trunk which controls the body's fight‐or‐flight response. This outpatient procedure, performed under ultrasound or fluoroscopic guidance, takes less than thirty minutes and is immediately effective. SGB is not a silver bullet; however, it has been used successfully for over ten years in conjunction with trauma‐focused psychotherapy to treat posttraumatic stress symptoms with a success rate of approximately 70%–80% (Lipov & Ritchie, 2015; Navaie et al., 2014). Due to its safety, effect size (symptoms scores reduced by 50%), and rapid onset of relief, SGB has gained wide acceptance in several locations including select US military hospitals where it has been available. In this time, SGB has been used effectively to help heal thousands of military service members suffering from symptoms associated with PTSD. A small sample of these cases is documented in the literature primarily in level 3 studies, yet if the collective results are considered in entirety, they show great consistency in SGB effects. There are 14 original studies published since 1990 in the peer‐reviewed medical literature documenting SGB’s successful treatment of PTSD symptoms (Alino, Kosatka, McLean, & Hirsch, 2013; Alkire et al., 2014; Alkire et al., 2015; Hanling et al., 2016; Lebovits, Yarmush, & Lefkowitz, 1990; Lipov, Joshi, Lipov, Sanders, & Siroko, 2008; Lipov et al., 2012, 2013; Lynch et al., 2016; McLean, 2015; Mulvaney et al., 2014; Mulvaney, Lynch, de Leeuw, Schroeder, & Kane, 2015; Mulvaney, McLean, & De Leeuw, 2010; Rae Olmsted et al., 2020). In November 2019, a large multicenter, randomized clinical trial demonstrated twice the effect of SGB over a sham procedure (Rae Olmsted et al., 2020).

Stellate ganglion block can be applied in terms of precision mental health for posttraumatic stress, not as a standalone treatment, but rather as an adjunct with a precise purpose to complement trauma‐focused psychotherapy. Patients treated with SGB report that many of their symptoms are improved, but particularly affected are hyperarousal symptoms such as irritability, angry outbursts, difficulty concentrating, and trouble falling or staying asleep (Lynch et al., 2016). For many of our patients, these dramatic improvements are life‐changing and enhance long‐term compliance with their psychotherapy. SGB improves more than just hyperarousal symptoms. While a decrease in intrusive memories, thoughts, and dreams has also been seen following this procedure, the most consistent improvement patients report following SGB is dramatic relief from undesired fight‐or‐flight reactions. (Lynch et al., 2016).

## 5 FUTURE DIRECTIONS

There are several unknowns regarding how to optimize SGB treatment for posttraumatic stress symptoms. For instance, when during therapy is SGB most effective? Targeting multiple levels of the cervical sympathetic trunk as well as the effects of right‐ versus left‐sided SGB have yet to be fully investigated. One possibility may include utilizing preprocedure fMRI to acquire laterality in amygdala hyperactivity in order to determine whether right‐ or left‐sided SGB is more appropriate. Preliminary work in this area shows promise (Kim, Park, Chung, & Kang, 2016). Future work should also attempt to determine the specific mechanism of action for SGB. Previous research has proposed a biologic mechanism related to brain norepinephrine levels (Lipov, Candido, & Ritchie, 2017; Lipov, Joshi, Sanders, & Slavin, 2009). While the precise mechanism of action for SGB is currently unclear, the evidence is convincing enough now to consider stellate ganglion block as an adjunct to trauma‐based psychotherapy for any patient exhibiting symptoms of increased hyperarousal.

## 6 CONCLUSION

Precision mental health treatments for posttraumatic stress disorder should (a) focus more on specific symptoms than a heterogenous diagnosis, (b) integrate emerging neuroscience with traditional models of mental illness, and (c) direct multidisciplinary, personalized therapies for individuals based on interventions that address their symptoms. Given these goals along with the presently expanding neurobiologic model of posttraumatic stress disorder, we can implement precision mental health now for potentially millions of patients by offering stellate ganglion block as part of their individualized treatment plan.

## CONFLICT OF INTEREST

The author has no conflicts of interests or has declared any such conflicts.

## Data Availability

Data sharing not applicable to this article as no datasets were generated or analysed during the current study.
